# Artificial Intelligence-Based Proximal Bone Shape Asymmetry Analysis and Clinical Correlation with Cartilage Relaxation Times and Functional Activity

**DOI:** 10.3390/bioengineering13020184

**Published:** 2026-02-05

**Authors:** Rafeek Thahakoya, Rupsa Bhattacharjee, Misung Han, Felix Gerhard Gassert, Johanna Luitjens, Valentina Pedoia, Richard B. Souza, Sharmila Majumdar

**Affiliations:** 1 Department of Radiology and Biomedical Imaging, University of California San Francisco, San Francisco, CA 94158, USA; 2 Department of Computer Science and Engineering, KMEA Engineering College, Edathala, Aluva 683561, India; 3 Department of Radiology, Klinikum Rechts der Isar, Technical University of Munich, Ismaninger Str. 22, 81675 Munich, Germany; 4 Department of Physical Therapy and Rehabilitation Science, University of California San Francisco, San Francisco, CA 94158, USA

**Keywords:** hip osteoarthritis, AI based automatic segmentation, bone shape asymmetry, functional activity, quantitative MRI, T_1rho_ and T_2_ relaxation time

## Abstract

The current study investigated proximal femur bone shape asymmetry and its associations with cartilage composition and functional performance in individuals with hip osteoarthritis (OA). Forty-seven participants with hip OA (mean age: 53.77 ± 12.47 years; 22 females; BMI: 24.49 ± 4.0 kg/m^2^) were included in this study. Bilateral hip MRI was performed using a 3.0 T MR scanner with 3D proton density fat-saturated CUBE and MAPSS sequences. Automatic segmentation of the proximal femur was achieved using a U-Net framework refined through a human-in-the-loop annotation strategy, followed by three-dimensional bone shape analysis to quantify asymmetry. Cartilage relaxation times were assessed using atlas-based segmentation and quantification, while functional activity was evaluated according to OARSI-recommended criteria. The proposed proximal femur bone segmentation showed a DSC of 96.48% (95%-CI: 96.33–96.64) and Hausdorff Distance of 4.66 mm (95%-CI: 3.80–5.51). Increased bone shape asymmetry in the posterior–lateral–superior region of the proximal femur was associated with functional activity in the chair stand test (rho = −0.41; *p* = 0.006), and the anterior–lateral–inferior region demonstrated a comparatively higher significant positive correlation (rho = 0.37; *p* = 0.006) with the $T_{1\mathrm{rho}\;}$values of the acetabular cartilage region. Overall, the findings indicate that region-specific proximal femoral bone shape asymmetry in hip OA is associated with cartilage characteristics and functional impairment, highlighting the potential value of bone shape features as imaging biomarkers relevant to clinical function.

## 1. Introduction

Hip osteoarthritis (OA) is the most common type of hip arthritis involving joint degeneration and is highly prevalent in the United States [1]. OA significantly affects the quality of life of patients and imposes substantial social and economic burdens [1,2]. Disease progression can be indicated by bone remodeling, the degeneration of articular cartilage, inflammation, and modifications to the overall joint structure. Increased bone remodeling occurs in early-stage OA subjects, whereas reduced bone remodeling and dense subchondral bone occur in the late stage of OA [3]. OA subchondral bone has an increased volume and thickness; however, it is weaker and less mineralized than normal bone [4]. Importantly, several studies have demonstrated bone shape changes in hip and knee joints in the early stage of OA [4,5,6].

Imaging-based quantitative assessments of proximal femur bone shape asymmetry are clinically important, as they enable objective detection of subtle structural variations between diseased and contra-lateral hips. In asymmetry studies, an asymmetric limb loading effect was observed while performing the sit-to-stand (STS) activity in subjects with weight-bearing asymmetry (WBA) in the late stage of hip OA before Total Hip Replacement (THR) [7,8]. In mild-to-moderate OA, during the phase of peak reaction force, OA patients unloaded the affected limb by transferring approximately 18.4% of their total load to the unaffected lower limb, while the performance in controls was symmetrical [9]. Additionally, proximal femur bone deformity in subjects with Legg–Calve–Perthes Disease (LCPD) or femoral acetabular impingement (FAI) increases the risk of developing early-stage OA [10,11]. Together, these findings highlight the importance of robust quantitative bone shape asymmetry analysis for gaining deeper insights into the joint structural changes associated with disease progression.

Recently developed high-resolution 3D isotropic MR imaging sequences may be useful for multiplanar reformatting, enhancing segmentation quality and reducing partial volume effects. Manual segmentation of bone shape images, often exceeding 150 images per bone, is tedious and prone to high inter-reader variability. To overcome this difficulty, automatic segmentation is required. Recently developed deep learning methods have shown promising results in hip bone shape segmentation [12,13,14,15,16]. Previously, bone shape analysis was performed based on quantifying the proximal femur geometry using 2D radiograph images [17,18,19]. In recent years, Statistical Shape Modeling (SSM) has been proven as a reliable method for shape analysis, especially in knee and hip OA subjects [5,20]. Additionally, bone shape analysis performed using SSM on MRI has shown better accuracy and reliability due to the superior resolution provided by MR images [21,22]. A recent study reported the association between 3D bone shape features and cartilage degeneration in OA subjects [23].

In addition to bone shape deformation, structural and biochemical changes in hip joint cartilage are strongly associated with the characteristics of OA disease [24]. The biochemical changes in articular cartilage can be evaluated using established compositional MRI techniques, including $T_{1\mathrm{rho}\;}$and $T_{2}$ relaxation time measurements [25,26]. Morphological changes in the cartilage typically occur in the later stage of the disease [4]. Previous studies have suggested that cartilage $T_{1\mathrm{rho}\;}$and $T_{2}$ measurements can serve as imaging biomarkers for early detection of OA [5,23].

Beyond structural and biochemical changes, hip OA causes pain and reduced joint mobility, which restricts physical activities in daily life [7,27]. In 2013, the Osteoarthritis Research Society International (OARSI) recommended five functional tests, including three minimal core set tests (30 s chair stand test (CST), stair climb test (SCT) and 40-m fast-paced walk test (FPWT)) to assess difficulties in daily activities for individuals with OA [28]. Therefore, a thorough investigation is needed to explore how asymmetries could be associated with biomechanical characteristics in individuals with hip OA.

We hypothesized that bone shape asymmetry is associated with hip cartilage abnormalities and the functional activities of OA subjects. The current study proposes a framework for automatic segmentation of proximal bone shape and investigates bone shape asymmetry and its association with biochemical changes in hip joint cartilage and functional activity measurements in subjects with hip OA.

## 2. Materials and Methods

### 2.1. Study Population

Subjects in the current study were part of a longitudinal study on hip OA. The well-established Kellgren–Lawrence (KL) grading system was employed for categorizing OA subjects. A KL score greater than one (KL score > 1) on any side of the hip (left or right) indicates OA, while a KL score of 1 or less classifies the control group [29]. The KL score was assessed by an experienced musculoskeletal radiologist (JL with 2 years of experience in musculoskeletal MR evaluation). In this study, subjects were included if they had a hip joint Kellgren and Lawrence score (KL score) less than 4. This study was approved by the local institutional review board. The inclusion criteria were as follows: age above 18, the absence of intra-articular injection in the past 6 months, no history of hip or knee surgery, and no contraindication for using MRI. Subjects fulfilling the criteria provided written informed consent and underwent imaging and functional activity experiments from December 2021 to July 2023.

### 2.2. Image Acquisition

MR images were acquired using a bilateral hip imaging protocol on a GE Signa Premier 3.0 T MR Scanner (GE Healthcare, Waukesha, WI, USA) utilizing a 30-channel adaptive image receive (AIR) anterior array coil and a 60-channel spine posterior array coil embedded into the table (GE Healthcare, Waukesha, WI, USA). The subjects were positioned supine with their feet slightly internally rotated and taped together to prevent movement during the scan. An isotropic 3D FSE (Fast Spin Echo) CUBE sequence and a magnetization-prepared angle-modulated Partitioned k-space spoiled gradient echo snapshot (MAPSS) sequence were employed for proximal femur bone shape analysis and cartilage $T_{1\mathrm{rho}\;}$and $T_{2}$ assessment, respectively. The detailed MR acquisition parameters used in the current study are described in Table 1.

### 2.3. Postprocessing of MRI Data

**Figure 1:**
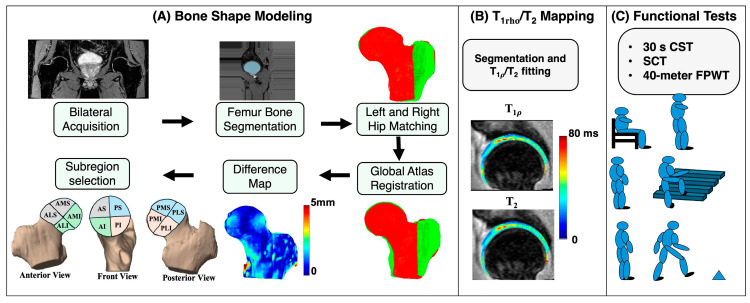
A schematic representation of the study methodology. (**A**) represents the bone shape modeling steps. (**B**) represents the cartilage segmentation results and $T_{1\mathrm{rho}\;}$ and $T_{2}$ map overlay. (**C**) represents the three core minimal functional activity tests recommended by Osteoarthritis Research Society International. AS: anterior superior; PS: posterior superior; AI: anterior inferior; PI: posterior inferior; AMS: anterior medial superior; AMI: anterior medial inferior; ALS: anterior lateral superior; ALI: anterior lateral inferior; PMS: posterior medial superior; PMI: posterior medial inferior; PLS: posterior lateral superior; PLI: posterior lateral inferior.

The postprocessing workflow was divided into three main components: bone shape segmentation and analysis, $T_{1\mathrm{rho}\;}$ and relaxation time quantification, and functional activity analysis. The overall processing pipeline is illustrated in Figure 1.

#### 2.3.1. Left and Right Hip Image Splitting

The 3D FSE CUBE sequence images were acquired in the coronal plane and reformatted into the sagittal plane, and left and right hip side image stacks were saved in separate directories using the GE SIGNA™ Works platform. From this point onwards, the left and right hip images from each subject were analyzed independently.

#### 2.3.2. Image Preprocessing

The preprocessing steps included data harmonization, normalization, and augmentation. Data harmonization was performed by selecting the first acquired data point as the target, comparing the field of view (FOV), matrix dimensions, and pixel spacing of all remaining datasets, and resizing the data to be comparable with the target data. All images were resampled to 512 × 512 using interpolation and the image background was padded with replication padding to preserve boundary information and avoid artificial artifacts. Image normalization was then carried out using a maximum percentile normalization approach. Finally, data augmentation was applied to improve model robustness, incorporating affine transformations along with the addition of various noise types.

#### 2.3.3. Bone Segmentation: Deep Learning-Based Approach

**Figure 2:**
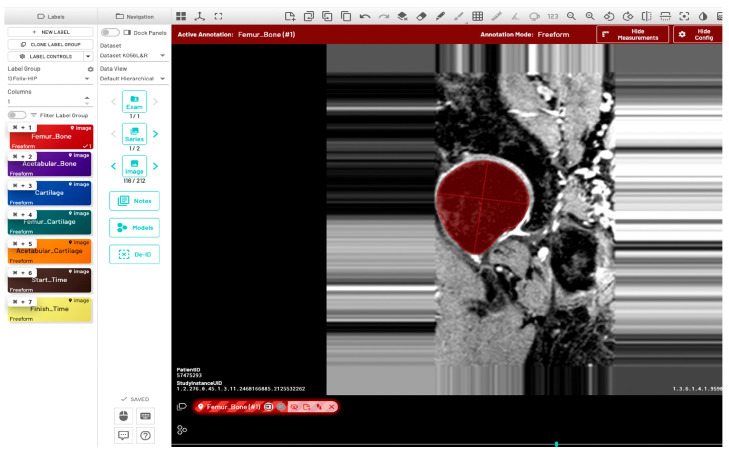
Annotation of proximal femur bone structure using the MD.ai software (MD.ai, New York, NY, USA https://md.ai/, accessed on 1 October 2023). The red circular area indicates the annotated region of the proximal femur bone shape.

Initially, a single dataset was manually segmented by two radiologists (FG and JL), both of whom have two years of experience in musculoskeletal MR image evaluation. The inter-reader variability between the two radiologists was quantitatively evaluated using the Dice Similarity Coefficient (DSC), Average Symmetric Surface Distance (ASSD), Relative Absolute Volume Difference (RAVD), and Hausdorff Distance (HD). Initial training of the model (model-1) utilized manually segmented ground-truth masks from five subjects (11%), with left and right hip annotations provided by two radiologists. Automatic bone segmentation was performed on the sagittal images using an in-house U-Net deep learning architecture [30]. All images were divided into training, validation, and test sets using a 70:15:15 split. Model training was performed using the following parameters: input image size = 512 × 512, learning rate = 0.001, optimizer = Adam, loss function = Binary cross entropy with logits, and number of classes = 1. Additionally, the pretrained checkpoints from a previously validated knee bone segmentation model [31] were utilized for model training. Automated predictions using the initial model were refined through a human-in-the-loop annotation strategy to generate ground-truth segmentations. The segmentation results of 32 subjects (68%) from model-1 were manually corrected by a single user (RT, trained by the radiologists) and verified by the radiologist JL. These masks were then used to fine-tune the model in a second round of training (model-2) with the following postprocessing methods: hole filling, morphological opening, and connected component analysis. The fine-tuned U-Net model was used to infer data for the final 10 subjects (21%). The inference of one data point was completed in approximately one minute. Manual segmentation and corrections were performed using the MD.ai (MD.ai, New York, NY) medical imaging platform (Figure 2).

#### 2.3.4. Bone Shape Analysis

**Figure 3:**
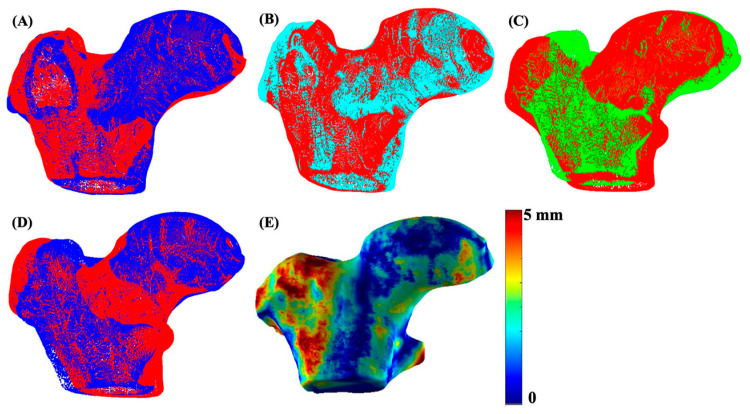
An illustration of 3D mesh formation, matching, and registration. Image (**A**) shows the 3D mesh overlay of the left (red) and right (blue) hip of a sample subject, image (**B**) shows meshes after matching and registration of the left (red) and right (cyan) hip using FOCUSR and ICP, image (**C**) shows the unregistered left hip mesh (green) with the global reference mesh (red), image (**D**) shows the meshes (blue) after global registration (ICP) with the reference mesh (red), and image (**E**) shows the final proximal femur bone shape difference map.

The 3D bone shape formation and quantitative analysis was performed using the in-house program developed with MATLAB (version R2023a, The MathWorks Inc., Natick, MA, USA). The segmented right proximal femur bone was flipped to match the orientation of the left hip. For a uniform shape structure, a femur bone shaft length cutoff was decided based on the minimum shaft length of the cohort and was applied to all subjects. The 3D meshes from all segmented stacks of images were generated using the MATLAB ‘isosurface’ function. The obtained triangular mesh was smoothened using Laplacian smoothing with inverse-vertices distance based umbrella weights [32]. The left bone mesh of each subject was aligned and matched with the already flipped right bone mesh, using the fast and precise FOCUSR surface matching method [32]. The iterative closest point algorithm (ICP) was then used for intra-subject rigid registration between the left and right hip for every patient. Once patient-level registration was complete, all bone meshes were subsequently aligned and registered with a reference atlas via the FOCUSR and ICP algorithms. The reference atlas was selected from the control group with the lowest KL score. Shape differences were quantified using the Euclidean distance between corresponding vertices of the registered three-dimensional (3D) meshes using the following formula:

(1) $$d\;=\;\sqrt{{\left(x_{2}-x_{1}\right)}^{2}+{\left(y_{2}-y_{1}\right)}^{2}+{\left(z_{2}-z\right)}^{2}}$$

where $(x_{1}$, $y_{1},\;z_{1}$) and $(x_{2}$, $y_{2},\;z_{2}$) represent the coordinates of corresponding vertices on the co-registered three-dimensional left and right meshes of each subject. The process for mesh matching and registration of the data for one subject is illustrated in Figure 3.

The diameter of the region of interest (ROI) for the femoral head region was selected as 55 mm based on the maximum diameter of a healthy femoral head [33]. The selected femoral head ROI was divided into eight equal subregions using the distance from point to plane method. The subregions were the anterior medial superior (AMS), anterior lateral superior (ALS), anterior medial inferior (AMI), anterior lateral inferior (ALI), posterior medial superior (PMS), posterior lateral superior (PLS), posterior medial inferior (PMI), and posterior lateral inferior (PLI).

#### 2.3.5. Cartilage $T_{1rho}$ and $T_{2}\;$ Quantification

$T_{1\mathrm{rho}\;}$and $T_{2}$ relaxation time-based quantification was performed using an in-house program developed in MATLAB integrated with the Elastix toolbox. After bilateral image acquisition, the left and right hip MAPSS sequence images in sagittal view were automatically divided into image stacks. $T_{1\mathrm{rho}\;}$and $T_{2}$ maps were obtained by fitting the multiple TSLs and TEs corresponding to the images using the Levenberg–Marquardt mono-exponential equation [34]. The hip cartilage regions were automatically segmented using a previously proposed and validated approach [35]. The first echo of $T_{1\mathrm{rho}\;}$ and $T_{2}$-weighted images referred to a previously defined single reference atlas using a non-rigid elastix registration method, and the registration transformation was applied to the remaining echo images. The atlas-based cartilage masks and sub-segmentations were developed using a semi-automated segmentation algorithm. The four slices near the hip center were selected as the ROI. The cartilage subregions are defined as follows: $R_{2}$ as posterior, $R_{3}$ as posterior–superior, $R_{4}$ as superior, $R_{5}$ as anterior–superior, $R_{6}$ as anterior, and $R_{7}$ as anterior–inferior. Six subregions were selected for the femur side ($R_{2}$–$R_{7}$) and five subregions ($R_{2}$–$R_{6}$) for the acetabular cartilage subregion analysis based on each region containing more than 50 pixels across all segmented slices [35]. A fixed threshold of 100 and 80 ms for $T_{1\mathrm{rho}\;}$ and $T_{2}$ maps was used to eliminate any possible fluid overlaps or partial volume effects during measuring [35].

### 2.4. Functional Activity Test

OARSI-recommended performance-based tests, including the 30 s CST, SCT, and 40-m FPWT, were used to assess the physical performance-based function of the subjects in this cohort [28]. During the CST, the participant’s arms were crossed over their chest, and the instructor counted the number of times they stood up and sat down on the chair in 30 s. In the SCT, the patient was instructed to walk up and down a flight of stairs, while the instructor measured the time taken to finish the task. In the 40-m FPWT, the patient was instructed to walk as quickly but as safely as possible without running to a cone 20 m away, turn around, and walk back at the same speed to the starting cone, with the time taken to complete the task recorded.

### 2.5. Statistical Analysis

The performance of automatic proximal femur bone segmentation was computed by using the DSC, ASSD, RAVD, and HD, expressed by the following equations:

(2) $$\mathrm{DSC}=\frac{2\left|V_{\mathrm{seg}}\cap V_{\mathrm{GT}}\right|}{\left|V_{\mathrm{seg}}\left|+\right|V_{\mathrm{GT}}\right|}$$

(3) $$\mathrm{ASSD}\;=\frac{1}{\left|B_{\mathrm{GT}}\right|+\left|B_{\mathrm{seg}}\right|}\times \left(\sum_{x\in B_{\mathrm{seg}}}d(x,{\;B}_{\mathrm{GT}})+\sum_{y\in B_{\mathrm{GT}}}d\left(y,{\;B}_{\mathrm{seg}}\right)\right)$$

(4) $$\mathrm{RAVD}\;=\frac{\left|\left|V_{\mathrm{seg}}\left|+\right|V_{\mathrm{GT}}\right|\right|}{\left|V_{\mathrm{GT}}\right|}\times 100$$

(5) $$\mathrm{HD}\;=\;\max\;\left(\begin{array}{c} max\\ x\in B_{\mathrm{seg}} \end{array}(d\left(x,B_{\mathrm{GT}})\right),\begin{array}{c} max\\ \;y\in B_{\mathrm{seg}} \end{array}(d\left(y,B_{\mathrm{GT}})\right)\right)$$

where $V_{\mathrm{seg}}$ is the mask developed by the proposed segmentation method and $V_{\mathrm{GT}}\;$ is the segmentation result after manual segmentation by the radiologists. ${\;B}_{\mathrm{GT}}$ and $B_{\mathrm{seg}}$ are the border voxel sets of the ground truth and segmented volume and d(x, y) is the Euclidean distance between voxels x and y.

Comparison of the control vs. OA group based on bone shape difference measurements (BSDMs) was performed using the unpaired *t*-test. All relevant statistical analyses were adjusted for age, gender, and body mass index (BMI) to account for potential confounding effects. For correlation analyses, cartilage degeneration and functional performance measures were obtained from the hip exhibiting greater disease severity. The Shapiro–Wilk test was performed to evaluate the normality of age, BMI, bone shape asymmetry measurements, cartilage $T_{1\mathrm{rho}\;}$and $T_{2}$ values, and functional activity results. A *p*-value of 0.05 was selected as the significant level. A *p*-value > 0.05 indicated normal distribution of the data, and Pearson’s correlation was used for the analysis. According to the Shapiro–Wilk test, *p*-value < 0.05 was observed in certain comparisons, indicating non-normal distribution; therefore, Spearman’s rank correlation was used for the analysis, which is less sensitive to outliers and extreme values. To control for multiple testing in the correlation analysis, the *p*-values reported in the table were adjusted using the Benjamini–Hochberg false discovery rate (FDR) correction [36]. Statistical analysis was performed using MATLAB and Microsoft^®^ Excel (Version 16.89) platforms.

## 3. Results

Forty-seven subjects (age = 53.77 ± 12.47 years; 22 females; BMI = 24.49 ± 4 kg/m^2^) were included in this study. Thirty subjects were labeled as controls (age = 48.70 ± 10.92 years; 14 females (47%); BMI = 23.72 ± 3.51 kg/m^2^), and 17 subjects were included in the OA group (age = 62.71 ± 9.91 years; 8 females (47%); BMI = 25.84 ± 4.53 kg/m^2^); 2 subjects were excluded because of poor segmentation results in the affected hip due to severe damage.

**Figure 4:**
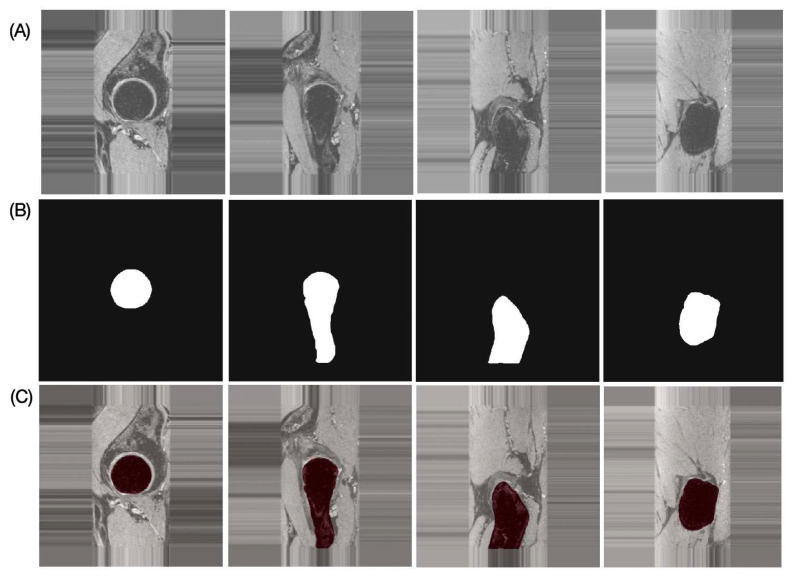
Representative proximal bone segmentation results for single subject. (**A**) Original images, (**B**) corresponding segmentation masks, and (**C**) segmentation results overlaid on original images.

The results of the performance evaluation metrics for inter-reader variability between FG and JL using the left and right hip side data of one subject with 180 images are as follows: DSC = 97.91%, RAVD = 1.43%, ASSD = 0.13 mm, and HD = 2.19 mm. The performance metrics of the proposed automatic bone segmentation method using the final model (model-2) in 10 subjects (20 hips), along with 95% confidence intervals, are as follows: DSC = 96.48% (95% CI: 96.33–96.64), RAVD = 1.13% (95% CI: 0.69–1.57), ASSD = 0.32 mm (95% CI: 0.29–0.35 mm), and HD = 4.66 mm (95% CI: 3.80–5.51 mm). Figure 4 presents representative automatic segmentation results of a selected patient (left hip) across selected slices.

**Figure 5:**
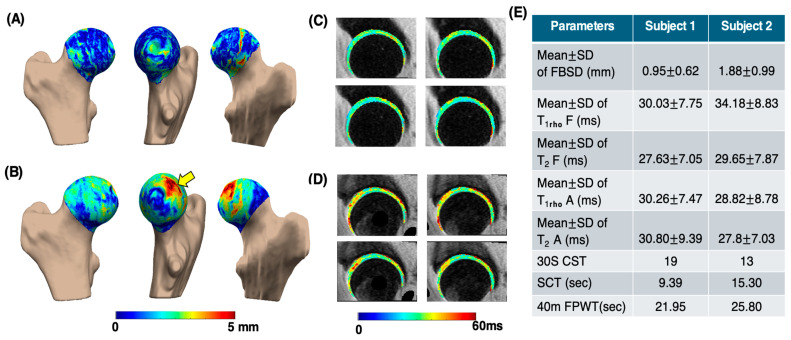
Sample comparison of various parameters between control and OA groups. Image (**A**,**B**) show femoral head bone shape difference maps for the control and OA subjects, respectively, highlighting differences in the posterior medial superior region (yellow arrow). Image (**C**,**D**) are the corresponding $T_{1\mathrm{rho}\;}$ (**first row**) and $T_{2}\;$ (**second row**) maps of the femur and acetabular cartilage, respectively. Image (**E**) shows the table consisting of different parameters. Mean ± SD of FBSD: mean ± SD of femoral head bone shape difference measurements. Mean ± SD of T_1rho_ F: T_1rho_ mean ± SD values of femur cartilage region. Mean ± SD of T_2_ F: T_2_ mean ± SD values of femur cartilage region. Mean ± SD of T_1rho_ A: T_1rho_ mean ± SD values of acetabular cartilage region. Mean ± SD of T_2_ A: T_2_ mean ± SD values of acetabular cartilage region.

Figure 5 presents the processing results of a sample subject from the control group (age: 66 years; gender: male; BMI: 23.17 kg/m^2^; and KL score 1 for left and 1 for right hip) and the OA group (age: 68 years; gender: female; BMI: 21.86 kg/m^2^; KL score 1 for left and 2 for right hip). In the OA subject, the PMS subregion exhibited the largest mean bone shape difference (3.64 ± 0.97 mm), whereas in the control subject, the greatest mean difference was observed in the PLI subregion (1.69 ± 1.12 mm). The elevated $T_{1\mathrm{rho}\;}$and $T_{2}$ maps of the femur and acetabular cartilage in the OA subject were compared to those of the control. Functional test results reflected the same trend, with the CST score for the control subject being 19, while the OA subject scored 13. This example indicates that OA subjects struggle to perform functional activities within the 30 s time limit. Additionally, the OA subjects took longer to complete tasks such as the SCT and 40-m FPWT.

**Figure 6:**
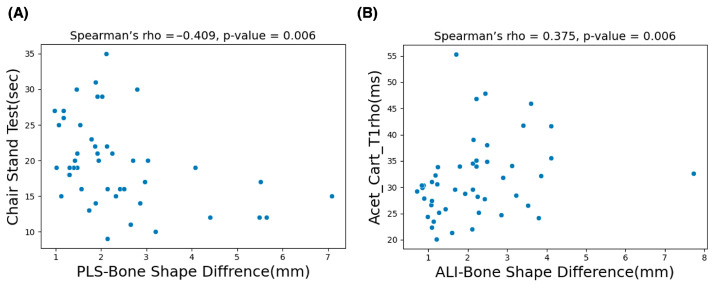
The scatter plots presented in (**A**) show the significant negative correlation results of the chair stand test (CST) and the mean values of bone shape difference measurements in the PLS (posterior–lateral–superior) subregion. The plots in (**B**) represent the significant positive correlation between mean $T_{1\mathrm{rho}\;}$values of the acetabular cartilage anterior region and the mean values of bone shape difference measurements in the ALI (anterior–lateral–inferior) subregion.

The G*Power version 3.1.9.6 sensitivity analysis results (α = 0.05, two-tailed; n_1_ = 30, n_2_ = 17) indicate that the study was sensitive for detecting large between-group effects (Cohen’s d ≈ 0.87). Table 2 shows the analysis results of control vs. OA group subjects based on BSDMs. In this comparison, the anterior lateral inferior and posterior medial superior region of the OA group exhibited significantly higher difference values (*p* = 0.043 and 0.037). Table 3 shows the correlation study results of all subjects. In this study, the BSDMs in the ALI region demonstrated a comparatively higher significant positive correlation (rho = 0.37; *p* = 0.006) with the $T_{1\mathrm{rho}\;}$values of the acetabular cartilage subregion. Moreover, the BSDMs in the PLS subregion exhibited a comparatively higher significant correlation (rho = −0.41; *p* = 0.006) with CST functional activity. Scatter plots of the variables with a significant correlation are shown in Figure 6.

## 4. Discussion

This study assessed the correlation of asymmetries—i.e., bone shape difference between the left and right hip—with cartilage health and functional activities in hip OA subjects. In this study, a U-Net-based automatic segmentation model was successfully developed by integrating a human-in-the-loop strategy with pretrained model checkpoints. The study results reveal a significant negative correlation between bone shape asymmetry in certain femur bone subregions with CST and SCT functional activities, indicating that hip OA subjects have difficulty in performing CST and SCT functional activities. The number of chair rises decreased in subjects with greater hip asymmetry, whereas OA subjects required more time to complete the stair climbing activity. Moreover, a greater extent of bone shape asymmetry was correlated with elevated hip femur and acetabular cartilage $T_{1\mathrm{rho}\;}$and $T_{2}$ relaxation times in different subregions. These findings support our hypothesis that individuals with OA exhibit greater bone shape asymmetry, elevated $T_{1\mathrm{rho}\;}$and $T_{2}$ relaxation time, and reduced functional performance.

Recently developed high-resolution isotropic 3D MRI acquisitions provide greater insight into disease progression, which can enhance clinical and research applications [37]. Acquiring isotropic images with sub-millimeter slice thickness and in-plane resolution potentially enhances the detailed evaluation of structural bone changes but also suffers from increased acquisition time. A previous feasibility study demonstrated that the acquisition of hip images of CUBE and MAPSS sequences using the bilateral acquisition method significantly reduced overall imaging time by 50.72% and 28.41%, respectively [38]. In our study, images of both hips were acquired simultaneously using the same bilateral image acquisition protocol. While the protocol benefits from higher image resolution, reducing the longer segmentation and analysis times due to dealing with many slices remains a challenge.

Fast and reliable automatic segmentation of bone structures is essential for accurate quantitative analysis of bone shape asymmetry. In this study, a U-Net-based deep learning framework was developed for the automatic segmentation of proximal femur bone structures. The bone shape segmentation model utilizing the U-Net architecture demonstrated a fast and reproducible segmentation process, and the performance evaluation metrics highlighted the excellence of the model in proximal femur bone shape segmentation. The incorporation of a human-in-the-loop strategy and pretrained model checkpoints further contributed to improved segmentation quality and model convergence, particularly in anatomically challenging regions. For the transfer learning strategy, a distal femur bone segmentation model trained on a large dataset and developed by our group [29] was utilized. This approach effectively addressed the limitations imposed by small sample sizes and minimized overfitting during model training. The human-in-the-loop approach further enhanced the reliability of the generated ground truth by combining automated predictions with expert radiologist verification.

Previously, plain radiographs were used to investigate the association of bone shape asymmetry with OA. The main drawback of radiographic studies is that they involve the analysis of bone shape features based on a 2D projection and, thus, cannot provide detailed information about the through-planes. The recently developed 3D statistical shape modeling (SSM)-based bone shape analysis using 3D MRI images provides a more accurate quantitative evaluation of bone shape due to higher resolution, the three-dimensional nature of images, and the improved accuracy of the algorithm [4,21,22]. These studies were reported in LCPD and FAI; those conditions are closely linked to early OA [10,11]. Therefore, analyzing bone shape asymmetry is essential for investigating the relationship between bone shape and other joint characteristics associated with OA. To the best of our knowledge, this is the first study to examine the correlation between bone shape asymmetry, biomechanics, and cartilage health.

OA subjects often experience decreased physical function and report challenges in daily activities. CST activity is considered a crucial activity in daily life, typically performed around 60 times a day by healthy individuals. It is particularly challenging for those with physical impairments and provides an effective quantitative measure of physical performance. Our results indicate that in hip OA subjects, BSDMs in the AMS, PMI, and PLS subregions were negatively correlated with CST functional activity. BSDMs in the femoral head region and AMS and PMI subregions were positively correlated with SCT functional activity. OA subjects required more time to complete the SCT, indicating greater difficulty in performing physical activities. In summary, the indication of asymmetry in OA subjects was shown by correlations between CST measurements and certain BSDM subregion values, as well as by the significant increase in task completion time during the SCT. The associations observed between bone shape asymmetry and functional outcomes should be interpreted within a multifactorial framework, as functional activities are influenced by a range of systemic factors, including muscle strength, balance, neuromuscular control, and cardiovascular fitness.

When comparing the control and OA groups, a 30% significant increase in the bone shape asymmetry values of subregions ALI and PMS were observed in OA subjects. Previous studies have reported that the entire articular surface of the acetabulum is involved in weight bearing. However, the contact areas of the femoral head are detected in the anterior, posterior, and superior regions [33,39], and no studies have mentioned the exact weight-bearing subregions in the femoral head. According to our results, anterior lateral bone shape asymmetry shows a significant positive correlation (rho = 0.37, *p* = 0.006) with the mean $T_{1\mathrm{rho}\;}$value of R_6_ (anterior) acetabular cartilage. The anterior subregion showed similar trends in bone shape asymmetry as well as changes in the biochemical properties of articular cartilage. Similarly, bone shape asymmetry showed a significant correlation with CST activity, with the most significant negative correlation found with the posterior side (rho = −0.41, *p* = 0.006). Table 4 summarizes MRI-based studies that evaluated hip joint cartilage composition using $T_{1\mathrm{rho}\;}$and $T_{2}$ mapping and functional biomechanics, highlighting how the present study uniquely links proximal femur bone shape asymmetry to cartilage relaxation changes and functional activity-related joint loading. Our findings suggest that patients with hip OA exhibit more specific regional changes in biochemical properties of the cartilage, greater differences in bone shape, and increased difficulty in performing functional activities compared to control subjects.

The diagnosis of hip dysplasia and femoral acetabular impingement (FAI) using MR images mainly depends on the quantitative assessment of proximal femoral asymmetry. These conditions are recognized as underlying causes of abnormal joint mechanics and are strongly associated with the later development of osteoarthritis (OA). However, in addition to proximal femoral bone asymmetry, acetabular bone morphology should be incorporated into quantitative evaluations of joint structure. Including both components can improve analysis of bone shape asymmetry and its association with cartilage deformation. As the hip joint functions as a dynamic system, its structural and pathological components are interdependent. Asymmetry in one component, such as deformation of the femoral head, may be functionally compensated by the morphology of the acetabulum, or vice versa. In addition, combined hip joint quantitative analysis enables more accurate estimation of joint contact area, stress concentration, load transfer, and asymmetric loading patterns that influence hip joint cartilage deformation. Therefore, incorporating both the proximal femur and acetabular regions in future studies may further enhance diagnostic accuracy and support improved treatment planning.

## 5. Limitations

Despite the promising results, the current study has limitations that need to be considered. While we only investigated the femur bone, hip joint loading also affects the shape of the acetabular bone. A shape assessment of the proximal femur and acetabular bone could provide a more reliable evaluation. The current study used a single imaging scanner and acquisition protocol and lacked an independent external validation cohort, which may limit generalizability. The applicability of these findings to other scanners, imaging protocols, or populations needs to be addressed in future studies. The relatively small sample size is a limitation of this study and may reduce sensitivity for detecting small effect sizes. However, the cohort size is comparable to previous MRI-based statistical shape modeling studies. Future studies with larger cohorts are required to confirm these findings and enable formal a priori power calculations. In this study, intra-reader variability was not assessed because, for large well-defined structures such as the proximal femur, repeated segmentation by the same reader typically shows excellent agreement using 3D DSC and other metrics; therefore, inter-reader variability is more informative and was thus emphasized in the present study. The relationship between age, gender, and BMI in relation to our observations was not included in the analysis due to the small sample size. In the current study, performance-based tests were used to assess physical function; a more detailed evaluation of kinetic and kinematic characteristics is required to observe the clear correlation between the loading effect and bone shape asymmetry and cartilage health of the proximal hip bone.

## 6. Conclusions

This study confirms that an automatic segmentation framework based on the U-Net architecture and transfer learning is a reliable approach for proximal hip bone segmentation. The results presented in this study confirm that the developed model using transfer learning reveals a significant relationship between proximal femur bone shape asymmetry and hip joint cartilage degeneration and functional activities. The study results confirm that the joint biomechanical loading effect is related to bone asymmetry and cartilage biochemical changes. Additionally, the results indicate that the superior regions of the femoral head are more greatly affected than other regions.

## Figures and Tables

**Table 1:** MRI parameters of bilateral acquisition protocol.

Parameters		
Scanner Used	GE Signa Premier 3.0 T MR Scanner (GE Healthcare, Waukesha, WI, USA)	
Coils Used	30-channel adaptive image receive (AIR) anterior array coil and 60-channel spine posterior array coil (GE Healthcare, Waukesha, WI, USA)	
Sequence Name	Hip MAPSS Sagittal	Fat-Suppressed 3D CUBE (Fast Spin Echo) Coronal
Acquisition Time	16 min 30 s	12 min 30 s
Acquisition Matrix	256 × 128	200 × 400
TR (per view)	5.2	1200
TSLs (ms)	0, 15, 30, 45	
TEs (ms)	0, 10.4, 20.8, 41.6	20.62
FOV (cm × cm)	14 × 14	16 × 32 (S/I × R/L)
Slice Thickness (mm)	4	0.8
ARC Acceleration Factor	2 × 2 (k_y_ × k_z_)	2 × 1 (k_y_ × k_z_)
Spin Lock Frequency	300 Hz	
Number of Slices	60	210–230

FOV: field of view; TR: recovery time; TSLs: spin lock times; TEs: echo times; MAPSS: magnetization-prepared angle-modulated partitioned k-space spoiled gradient echo snapshot.

**Table 2:** Mean bone shape difference measurements (BSDMs) in femoral head and subregions between control and OA groups. * indicates *p*-value < 0.05.

BSDM (mm) (Mean ± SD)	Head	AMS	AMI	ALS	ALI	PMS	PMI	PLS	PLI
Control Group	2.11 ± 0.79	2.12 ± 1.11	2.03 ± 0.77	2.04 ± 1.06	1.90 ± 0.84	2.03 ± 1.18	2.26 ± 1.77	2.30 ± 1.37	2.26 ± 1.53
OA Group	2.54 ± 0.88	2.43 ± 1.15	2.52 ± 1.11	2.49 ± 1.44	2.68 ± 1.73	2.92 ± 1.64	2.87 ± 1.31	2.40 ± 1.34	2.53 ± 1.48
*p*-value	0.097	0.371	0.079	0.235	0.043 *	0.037 *	0.221	0.811	0.548

Head: proximal femur head region; AMS: anterior medial superior; AMI: anterior medial inferior; ALS: anterior lateral superior; ALI: anterior lateral inferior; PMS: posterior medial superior; PMI: posterior medial inferior, PLS: posterior lateral superior; PLI: posterior lateral inferior.

**Table 3:** Significant correlation between bone shape difference measurements (BSDMs) with cartilage mean $T_{1\mathrm{rho}\;}$ and T_2_ measurements and functional activities.

Parameters	*p*-Value	R/rho-Value	Correlation Type
Head vs. T_2_ femur R_3_	0.047	0.30	Pearson
ALI vs. T_2_ acetabular R_6_	0.047	0.19	Pearson
$\mathrm{ALI}\;\mathrm{vs}.\;T_{1\mathrm{rho}\;}$ femur R_2_	0.039	0.32	Spearman Rank
AMI vs. T_2_ femur R_3_	0.039	0.32	Spearman Rank
ALS vs. T_2_ femur R_3_	0.039	0.31	Spearman Rank
PMI vs. T_2_ femur R_3_	0.042	0.30	Spearman Rank
$\mathrm{ALI}\;\mathrm{vs}.\;T_{1\mathrm{rho}\;}$ acetabular R_5_	0.049	0.29	Spearman Rank
$\mathrm{ALI}\;\mathrm{vs}.\;T_{1\mathrm{rho}\;}$ acetabular R_6_	0.006	0.37	Spearman Rank
PMS vs. T_2_ acetabular R_6_	0.042	0.30	Spearman Rank
PMI vs. T_2_ acetabular R_6_	0.039	0.31	Spearman Rank
AMS vs. CST	0.039	−0.31	Spearman Rank
PMI vs. CST	0.039	−0.34	Spearman Rank
PLS vs. CST	0.006	−0.41	Spearman Rank
Head vs. SCT	0.039	0.33	Spearman Rank
AMS vs. SCT	0.039	0.34	Spearman Rank
PMI vs. SCT	0.039	0.30	Spearman Rank

*p*-values after FDR correction; Head: proximal femur head region; AMS: anterior medial superior; AMI: anterior medial inferior; ALS: anterior lateral superior; ALI: anterior lateral inferior; PMS: posterior medial superior; PMI: posterior medial inferior; PLS: posterior lateral superior; PLI: posterior lateral inferior; R_2_: posterior; R_3_: posterior–superior; R_5_: anterior–superior; R_6_: anterior; CST: chair stand test; SCT: stair climb test.

**Table 4:** Summary of different quantitative hip bone shape asymmetry studies and corresponding findings.

Study	Anatomical Focus	Population	Assessment Method	Quantitative Metrics	Key Findings
Harris et al., 2012 [11]	Proximal femur and acetabulum	Healthy subjects	CT	Contact area, Load transfer and stress concentration	Hip contact stresses are concentrated in anterior–superior regions during weight bearing
Farkas et al., 2015 [40]	Proximal femur	FAI Patients	Radiographs and Gait analysis	Alpha angle, Cener edge angle Gait kinetics/kinematics	Cam morphology associated with altered hip kinematics
Valentina et al., 2016 [5]	Proximal femur and acetabulum	Healthy and OA subjects	MRI and T_2_ mapping	T_1rho_ and T_2_ relaxation times	Early cartilage matrix degeneration detected using MRI prior to radiographic OA
Subburaj et al., 2013 [41]	Proximal femur and acetabulum	Healthy and FAI Patients	MRI and T_2_ mapping	T_1rho_ and T_2_ relaxation times	Anterior-superior cartilage sub-region of patient were significantly different from controls
Youssefian et al., 2021 [42]	Proximal Femur	Cadaver	CT-based FE Models	Susceptibility of Stress	The model was more susceptible to element size and density–elasticity relationships
Proposed study	Proximal Femur	Health and OA subjects	MRI and T_2_ mapping, bone shape asymmetry and functional analysis	Bone shape difference measurements, T_1rho_ and T_2_ relaxation times and functional activity parameters	Bone shape asymmetry correlated with biochemical characteristic changes of cartilage and functional activities

## Data Availability

The data presented in this study are only available on request from the corresponding author, as they are part of an ongoing project.
